# Frog Skin Microbiota Vary With Host Species and Environment but Not Chytrid Infection

**DOI:** 10.3389/fmicb.2020.01330

**Published:** 2020-06-24

**Authors:** Ariel Kruger

**Affiliations:** Department of Ecology, Evolution, and Natural Resources, Rutgers University, New Brunswick, NJ, United States

**Keywords:** amphibians, *Batrachochytrium dendrobatidis*, chytrid fungus, disease, host-pathogen, skin microbiota

## Abstract

Describing the structure and function of the amphibian cutaneous microbiome has gained importance with the spread of *Batrachochytrium dendrobatidis* (Bd), the fungal pathogen that can cause the skin disease chytridiomycosis. Sampling amphibian skin microbiota is needed to characterize current infection status and to help predict future susceptibility to Bd based on microbial composition since some skin microbes have antifungal capabilities that may confer disease resistance. Here, I use 16S rRNA sequencing to describe the composition and structure of the cutaneous microbiota of six species of amphibians. Frog skin samples were also tested for Bd, and I found 11.8% Bd prevalence among all individuals sampled (*n* = 76). Frog skin microbiota varied by host species and sampling site, but did not differ among Bd-positive and Bd-negative individuals. These results suggest that bacterial composition reflects host species and the environment, but does not reflect Bd infection among the species sampled here. Of the bacterial OTUs identified using an indicator species analysis as strongly associated with amphibians, significantly more indicator OTUs were putative anti-Bd taxa than would be expected based on the proportion of anti-Bd OTUs among all frog OTUs, suggesting strong associations between host species and anti-Bd OTUs. This relationship may partially explain why some of these frogs are asymptomatic carriers of Bd, but more work is needed to determine the other factors that contribute to interspecific variation in Bd susceptibility. This work provides important insights on inter- and intra-specific variation in microbial community composition, putative function, and disease dynamics in populations of amphibians that appear to be coexisting with Bd.

## Introduction

The bacterial symbionts on amphibian skin can improve disease outcomes resulting from exposure to a fungal pathogen, *Batrachochytrium dendrobatidis* (Bd) (Woodhams et al., 2007; Harris et al., 2009; Becker and Harris, 2010), which can cause the disease chytridiomycosis. This disease is implicated as the cause of the unprecedented loss of amphibian biodiversity that has occurred globally in recent decades (Scheele et al., 2019). Understanding the composition of amphibian cutaneous microbial communities is increasingly important with the spread of Bd. For instance, the presence of cutaneous symbiotic microbes with anti-Bd activity has been linked to Bd resistance in some populations (Woodhams et al., 2007). In addition, the recent increase in assessments of host-associated microbiota in wild populations of frogs (Belden et al., 2015; Rebollar et al., 2016b; Bletz et al., 2017a) and salamanders (Muletz-Wolz et al., 2017b; Bird et al., 2018) has provided valuable evidence demonstrating the role of amphibian skin microbes as agents of disease resistance.

Previous studies of amphibians have shown that many skin microbial communities are specific to particular host species (e.g., McKenzie et al., 2012; Kueneman et al., 2014; Walke et al., 2014). The composition of amphibian-associated cutaneous microbes can be determined by both intrinsic factors such as host immunity and frequency of skin shedding, and extrinsic factors, such as environmental temperature and pathogen presence (Rebollar et al., 2016a). Interspecific variation in skin microbiota is a proposed mechanism for differences in Bd susceptibility (McKenzie et al., 2012) although a combination of factors, such as production of antimicrobial peptides on the skin (Myers et al., 2012) and the adaptive immune system (Ramsey et al., 2010) likely influence disease susceptibility. Intraspecific variation in Bd susceptibility can also exist, with different populations of the same species showing varying responses to Bd due to population-level differences in the presence of anti-Bd skin microbes (Woodhams et al., 2007).

The pool of microbial taxa present in the local environment may also contribute to population-level differences in Bd susceptibility. Previous evidence suggests that the environment plays an important role in determining the microbial community structure of amphibian skin (Muletz-Wolz et al., 2017b; Jani and Briggs, 2018), possibly by acting as a reservoir from which potential skin microbes are recruited (Loudon et al., 2014). Furthermore, anti-Bd bacterial taxa can vary among localities (Flechas et al., 2012; Muletz-Wolz et al., 2017a; Kruger, 2019), and the prevalence of Bd-inhibitory bacteria may be related to environmental factors such as soil pH (Varela et al., 2018) or temperature (Muletz-Wolz et al., 2019). Taken together, these findings suggest that the local environment can mediate the presence and prevalence of anti-Bd microbes on amphibian skin and may therefore partially explain differences in Bd susceptibility among populations.

In addition to the influence of the local environment, infection with Bd can conceivably alter the diversity and structure of amphibian skin microbial communities (Jani and Briggs, 2014; Rebollar et al., 2016b). Disruption of normal microbial functioning from other stressors, such as environmental pollutants (Kohl et al., 2015), can also affect host health and disease progression (Hamdi et al., 2011; Thomason et al., 2017). Because disruption from Bd infection and from external stressors that leads to Bd infection both manifest as differences in microbial community structure in assessments of wild amphibian popualtions, it can be difficult to infer cause and effect when examing differences in microbiota among infected and noninfected individuals. Additionally, these responses are not mutually exclusive (Jani and Briggs, 2014; Walke et al., 2015). Through sampling pre-epizootic populations, Jani et al. (2017) were able to demonstrate that Bd establishment results in changes to bacterial community structure on amphibian skin. Regardless of the direction, there seems to be a clear connection between Bd infection and microbial community structure, with convincing evidence that Bd infection is capable of causing changes in the bacterial portion of amphibian skin microbiomes. Because of interspecific differences in both host microbiota and Bd susceptibility, more work is needed to understand how the realtionship between Bd and skin microbiota varies among species.

Bd has been present in North America since as early as 1888 (Talley et al., 2015) and is widespread in northeastern states (Longcore et al., 2007; Richards-Hrdlicka et al., 2013; Julian et al., 2019). However, there is little evidence of population declines due to Bd in this area (Longcore et al., 2007; Richards-Hrdlicka et al., 2013). Current climatic conditions may contribute to the present lack of lethality in this region because of seasonal variations in Bd prevalence (Julian et al., 2016). However, it is possible that environmental changes associated with climate change could trigger future lethal outbreaks of disease (Rohr and Raffel, 2010; Cohen et al., 2017). The widespread prevalence of Bd in this region is concerning given the context-dependent nature of this host-pathogen interaction. As such, documenting current infection prevalence may help determine which species are most at risk of future declines.

In this study, I describe the composition, predicted anti-Bd function, and Bd status of frogs’ skin microbial communities across several localities in New Jersey. There has been some evidence of Bd in New Jersey (Monsen-Collar et al., 2010; Chu et al., 2014) but more work is needed to comprehensively determine Bd prevalence in the state. Using culture-independent methods, the objectives of this study were to (i) characterize the composition and diversity of skin bacterial communities of frogs at several sites in New Jersey, (ii) compare amphibian skin microbiota to environmental bacterial communities, (iii) determine whether Bd infection is associated with differences in skin microbiota, and (iv) examine the prevalence of potentially Bd*-*inhibiting bacteria among species and sites.

## Materials and Methods

### Field Sampling

I sampled the cutaneous microbiota of spring peepers (*Pseudacris crucifer*), wood frogs (*Lithobates sylvaticus*), green frogs (*Lithobates clamitans*), bullfrogs (*Lithobates catesbeianus*), carpenter frogs (*Lithobates virgatipes*), and Pine Barrens treefrogs (*Hyla andersonii*) across five sites: Success Lake in Colliers Mills Wildlife Management Area (WMA), Jackson, NJ, Albertson Bog in Wharton State Forest, Hammonton, NJ, Morin Pond and Kai Pond in Somerset, NJ, and Imlaystown Bog in Assunpink WMA, Allentown, NJ (Supplementary Figure S1). Frog species were selected based on preliminary visual surveys, such that there was a sufficiently large population at each site to make sampling feasible. Sites were further selected based on permit regulations and pond accessibility. Two species – green frogs and bullfrogs – were sampled at more than one site, and only green frogs were sampled at more than two sites. Frogs were sampled in the spring and summer of 2016 and 2017, and I aimed to sample 10 individuals of target species at each site (Table 1). Sampling methods were as previously described (Kruger, 2020). Briefly, each frog was handled with new nitrile gloves and rinsed twice with sterile deionized water to exclude transient matter that is not part of the skin-associated microbial community (McKenzie et al., 2012; Kueneman et al., 2014). I swabbed each frog with a sterile cotton swab (Medline MDS202000) 20 times (five streaks each on the dorsal side, ventral side, and each hind limb). All amphibians were released immediately after sampling. Environmental bacteria were sampled by swirling a swab in pond water at approximately 10 cm depth for 5 s (*n* = 9 across sites, Table 1). Swabs were placed in sterile centrifuge tubes on ice and subsequently preserved at −80°C until DNA extraction. The New Jersey DEP approved this protocol for sampling amphibian skin bacteria (NJDEP Scientific Collecting Permit Nos. SC 2016093 and 2017053), and amphibian sampling methods were approved by Rutgers University’s Institutional Animal Care and Use Committee (Protocol #14-080).

**TABLE 1:** Summary of frog and environmental sampling by site.

Site	Sample origin	Date sampled	*N* (*N* after filtering)	Bd-positive
Assunpink WMA				
	Bullfrog	5/22/17	10 (10)	2
	Green frog	7/2/16	10 (10)	1
	Environment	5/22/17, 7/22/17	2 (2)	–
Kai Pond				
	Bullfrog	8/24/17	3 (3)	0
	Spring peeper	4/25/17	7 (7)	4
	Environment	4/25/17, 8/24/17	2 (2)	–
Morin Pond				
	Green frog	5/13/16	10 (9)	0
	Wood frog	3/9/16	10 (10)	2
	Environment	5/13/16, 3/9/16	2 (2)	–
Colliers Mills WMA				
	Carpenter frog	7/27/17	10 (8)	0
	Green frog	7/7/16	10 (10)	0
	Environment	7/7/17 (7/27/17)	2 (1)	–
Albertson Bog				
	Pine Barrens treefrog	4/30/17	10 (9)	0
	Environment	4/30/17	1 (1)	–

*“N” indicates the number of individuals swabbed from each population, while the “N” in parentheses includes the number of individuals retained in analyses after quality control filtering during bioinformatics. “Bd-positive” indicates the number of samples that tested positive for Bd in qPCR assays. Environmental samples were not tested for Bd.*

### 16S rRNA Amplicon Sequencing

I extracted DNA from swabs using the Qiagen DNeasy PowerSoil kit following kit instructions with modifications according to the Earth Microbiome Project for low DNA amounts (Berg-Lyons et al., 2018). After extraction, an initial round of PCR of the full 16S gene with 27F and 1492R primers was performed to deal with low DNA concentrations. Each 20 μL reaction contained 0.02 U/uL Phusion^®^ DNA polymerase, 200 μM dNTPs, 0.5 μM forward primer, 0.5 μM reverse primer, and 1 μL DNA template. The mixtures were amplified at 98°C for 30 s, followed by 35 cycles of 98°C for 10 s, 62°C for 30 s, 72°C for 45 s and a final elongation for 7 min. I checked products for successful amplification using gel electrophoresis. PCR products were cleaned using the AxyPrep Mag PCR cleanup kit per kit instructions and sent to the Integrated Microbiome Resource lab at Dalhousie University (Halifax, Canada) for library preparation and sequencing. A second round of PCR of the V4-V5 region using the 515F and 926R primers was performed, and pooled PCR amplicons were sequenced by an Illumina MiSeq using 2x300 bp paired-end v3 chemistry. Sequencing procedural details were as described by Comeau et al. (2017).

### Sequence Processing

Sequence assembly was performed by the Integrated Microbiome Resource lab at Dalhousie University according to the Microbiome Helper standard operating procedures workflow (Comeau et al., 2017) using QIIME v. 1.9.1 (Caporaso et al., 2010). Briefly, sequences were clustered into operational taxonomic units (OTUs) based on 97% sequence similarity using open-reference OTU picking with QIIME wrapper scripts (Caporaso et al., 2010), and taxonomy was assigned using the Greengenes database (DeSantis et al., 2006). Sequences without taxonomic matches were clustered *de novo* at the 97% sequence similarity level. Sequences were quality filtered such that low-confidence OTUs making up <0.1% of reads and chimeric reads were removed. Sequencing depth per sample ranged from 530 to 77,737 reads. I rarefied all samples to a depth of 2500 reads to standardize sampling effort while maximizing sample inclusion, and samples that did not reach this threshold (*n* = 5) were removed from subsequent analyses. After filtering, 76 frog skin samples and 8 environmental samples remained (Table 1). Sequences have been deposited in the SRA database (Bioproject accession number: PRJNA601697).

### Bd Testing

I sent extracted DNA to Pennsylvania State University-Altoona College for Bd-testing using qPCR, where primers developed by Boyle et al. (2004) were used. All samples were run in triplicate with positive and negative controls using qPCR conditions described by Julian et al. (2016). A synthetic gBlock^®^ fragment of the ITS-1 and 5.8 ribosomal genes of Bd (GenBank accession number AY598034) was used to generate a standard curve ranging from 9 to 9000 zoospore equivalents to allow estimation of Bd concentrations. Individuals were considered Bd-positive if at least two out of three qPCR wells were positive (Kerby et al., 2013; Richards-Hrdlicka et al., 2013; Becker et al., 2015a) at a detection threshold of >1.0 zoospore equivalents (Supplementary Table S1). If one of the three wells was positive, the reaction was run in triplicate again. If at least one of the wells was positive on the second run of PCR, this was considered a positive detection of Bd. Bd infection intensity for each sample was calculated as log-transformed mean zoospore equivalents across technical (positive) replicates.

### Bacterial Community Diversity Metrics

Shannon index, Chao1, Faith’s phylogenetic distance (Faith, 1992), and observed OTU richness alpha diversity metrics were calculated with QIIME (Caporaso et al., 2010). Beta diversity was calculated using weighted and unweighted Unifrac distances (Lozupone et al., 2011), Bray–Curtis dissimilarity (Bray and Curtis, 1957), and Jaccard indices in the phyloseq package (McMurdie and Holmes, 2013) in R version 3.5.1 (R Core Team, 2016). Unifrac metrics encompass information on phylogenetic relatedness (Lozupone et al., 2011), while Jaccard and Bray–Curtis do not. Jaccard and unweighted Unifrac use only a presence/absence matrix, while Bray–Curtis and weighted Unifrac use a matrix containing relative abundances of OTUs sampled on each individual. I visualized results using non-metric multi-dimensional scaling (NMDS).

I compared the bacterial OTUs identified in this study to OTUs in a published database containing bacterial isolates known to inhibit or enhance Bd activity (Woodhams et al., 2015). I used a custom blast search in Geneious version 11.1.3 (Kearse et al., 2012) to find which potentially inhibitory Bd OTUs were present in my dataset based on >97% sequence similarity. Sequence similarity does not necessarily imply Bd-inhibitory function, but rather suggests this function is likely (Muletz-Wolz et al., 2017a). I used the megablast tool and had Geneious return only the top hit from the antifungal isolate database (Muletz-Wolz et al., 2017b).

### Indicator Analysis

I conducted indicator species analyses using the “multipatt” function in the indicspecies package (De Cáceres and Legendre, 2009) in R (R Core Team, 2016). This analysis identifies species that characterize a group of sites, or in this case, bacterial OTUs that characterize different frog species, based on their relative abundance and frequency of occurrence (Dufrene and Legendre, 1997). OTU tables were filtered before analyses to exclude environmental bacterial samples, thereby only including amphibian-associated bacteria. I identified OTUs that had high association values (IndVal > 0.7, Becker et al., 2017) and were significantly (*p* < 0.05) associated with host species. I also performed an indicator analysis between Bd positive and negative individuals to determine which OTUs were strongly associated with individuals based on Bd status. I compared the indicator OTUs to the antifungal isolate database (Woodhams et al., 2015) to determine how many indicator OTUs were putative anti-Bd bacteria.

### Statistical Analysis

All statistical analyses were performed using R (R Core Team, 2016). I analyzed the effect of host species identity on each alpha diversity metric with a mixed effects model using the lme4 package (Bates et al., 2015). Host species was assessed as a fixed effect, site was included as a random effect, and significance of effects was determined using the “Anova” function in the car package (Fox et al., 2016). I used Shapiro–Wilk tests to assess normality and evaluated model fit using residual plots. Chao1 index data were square-root transformed and analyzed with a linear mixed effects model, and OTU richness data were analyzed using a generalized linear mixed effects model with a negative binomial error distribution. I used Kruskal–Wallis tests followed by FDR-corrected Wilcoxon tests to determine if bacterial community alpha diversity varied based on Bd status (positive vs. negative individuals) and sample type (frog vs. environment). Because green frogs were the only species sampled at more than two sites, I analyzed patterns across these populations to determine the effect of sampling site on alpha diversity using Kruskal–Wallis tests.

I determined the effect of host species on cutaneous bacterial beta diversity using permutational multivariate analysis of variance (PERMANOVA) with the “adonis” function (999 permutations) in vegan (Oksanen et al., 2013). When testing the importance of frog species in influencing community beta diversity, the “strata” argument accounted for sampling site. A separate PERMANOVA was used to compare beta diversity between Bd-positive and Bd-negative individuals. As described above, I analyzed patterns in the three green frog populations to determine the effect of sampling site on bacterial community structure. I also used a PERMANOVA to compare beta diversity between environment and frog-associated bacterial communities. Pairwise PERMANOVAs compared significant effects. I used the false discovery rate (FDR) procedure to correct for multiple comparisons (Benjamini and Hochberg, 1995). The “betadisper” function in vegan (Oksanen et al., 2013) was used to run tests for homogeneity of dispersions for frog species and sites (using green frog populations), and Tukey’s HSD test was used for *post hoc* analyses. Only significant betadisper results are reported, since heterogeneous dispersion among groups can influence PERMANOVA results.

To analyze the diversity (i.e., number and total relative abundance) of potentially anti-Bd bacteria, I used mixed effects models with Bd status and host species as fixed effects and site as a random effect. Among the green frogs, I used Kruskal–Wallis tests to determine if the number and total relative abundance of putative anti-Bd OTUs varied across sites. I compared the proportion of putative anti-Bd OTUs among all frog-associated OTUs to the proportion of these OTUs among indicator taxa using a Chi-square goodness of fit test to determine if more indicator OTUs were inhibitory than expected based on their prevalence among all frog-associated OTUs.

## Results

**FIGURE 1:**
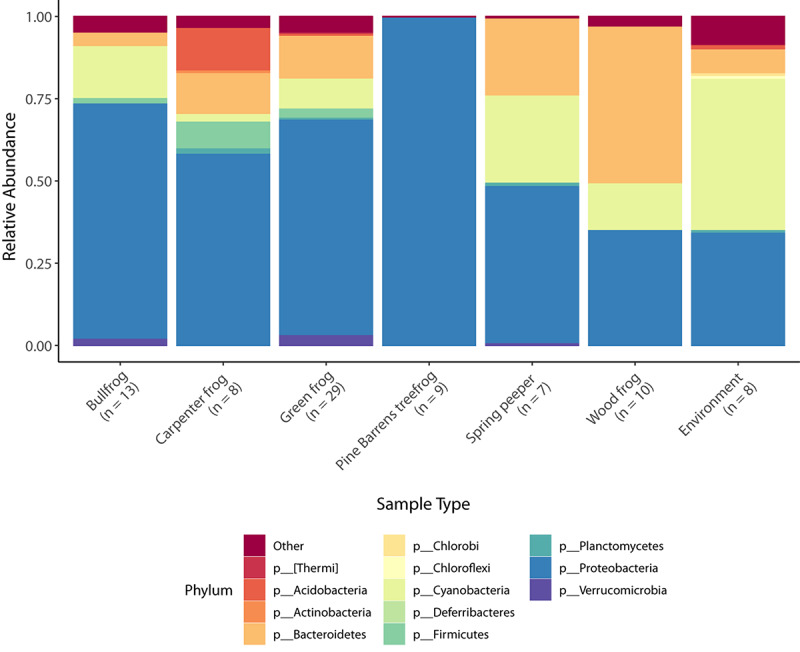
Taxonomic bar plots of mean relative abundance of bacteria (phylum level) across species and in the environment. Phyla comprising less than 5% relative abundance are grouped together in the “other” category.

There were a total of 2371 bacterial OTUs representing 24 phyla identified in frog and environmental bacterial samples. The five most abundant phyla present in frog skin samples by percent composition were Proteobacteria, Bacteroidetes, Cyanobacteria, Firmicutes, and Acidobacteria (Table 2), and the dominant phylum differed among frog species and the environment (Figure 1).

**TABLE 2:** Phylum-level summary of OTU distribution in frog bacterial community samples.

Phylum	Proportion OTUs	Mean relative abundance
p__Proteobacteria	0.536	0.635
*c__Alphaproteobacteria*	*0.145*	*0.063*
*c__Betaproteobacteria*	*0.306*	*0.481*
*c__Gammaproteobacteria*	*0.062*	*0.087*
*c__Deltaproteobacteria*	*0.021*	*0.005*
*c__Other*	*0.003*	*0*
p__Bacteroidetes	0.177	0.157
p__Cyanobacteria	0.064	0.110
p__Firmicutes	0.057	0.025
p__Acidobacteria	0.042	0.018
p__Actinobacteria	0.024	0.004
p__Planctomycetes	0.021	0.008
p__Verrucomicrobia	0.015	0.022
p__Chloroflexi	0.006	0.001
p__Armatimonadetes	0.005	0.002
p__Gemmatimonadetes	0.005	0.001
p__[Thermi]	0.004	0.002
p__Chlorobi	0.003	0.001
p__OP3	0.001	0.001
p__GN02	0.001	0.001
p__Deferribacteres	0	0.001
Unclassified	0.030	0.011

*Proportion of OTUs was calculated out of the total number of OTUs found on frog skin across samples (*n* = 2194). Mean relative abundance is across all individuals sampled, regardless of species or site (*n* = 76). Proteobacteria made up the majority of OTUs in the dataset and are further broken down by class. Phyla with less than 0.1% relative abundance are excluded.*

### Alpha Diversity

**FIGURE 2:**
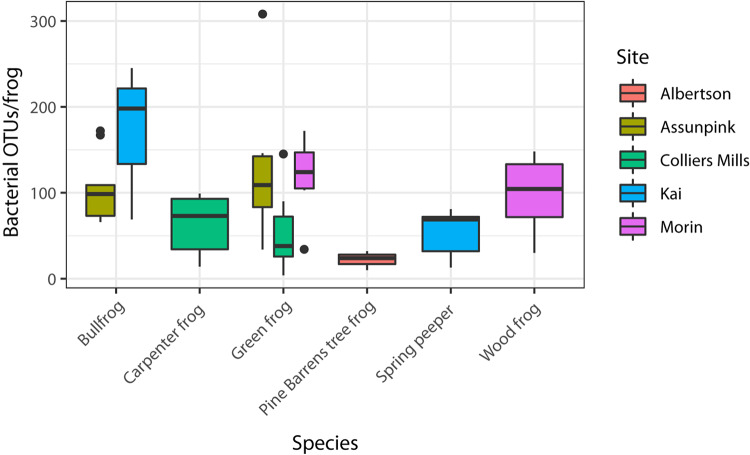
Alpha diversity (OTU richness) varied among the six host species sampled (GLMM *p* < 0.05). OTU richness also varied significantly (KW *p* < 0.05) among the three green frog populations, which were used to assess site-level variation in alpha diversity because they were the only species sampled at more than two sites.

All alpha diversity metrics yielded similar results. Hence, here I present the diversity estimates of Chao1, a metric commonly used for measuring microbial diversity, and OTU richness. OTU richness was variable across individuals, ranging from 4 to 312 OTUs per frog. OTU richness (Figure 2; GLMM: χ^2^ = 22.68, df = 5, *p* < 0.001) and Chao1 index (LMM: χ^2^ = 11.54, df = 5, *p* = 0.042) varied significantly among host species. Among green frogs, the only frog species sampled at more than two sites, there were significant differences in alpha diversity (KW – OTU richness: χ^2^ = 10.02, *p* = 0.007; Chao1: χ^2^ = 10.29, *p* = 0.006) among populations. These results indicate that both host species and sampling location influenced individual-level bacterial community diversity.

### Beta Diversity

**FIGURE 3:**
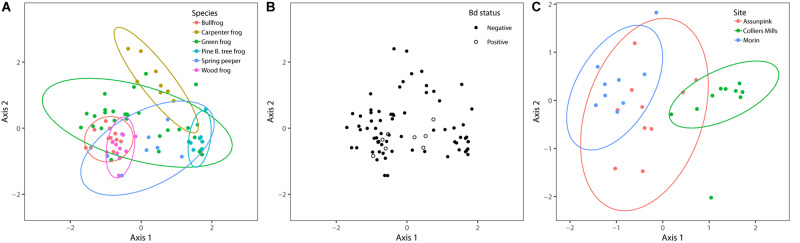
NMDS plots of bacterial communities based on Bray–Curtis distances and grouped by **(A)** frog species, **(B)** Bd status, and **(C)** sampling site. Each point represents an individual amphibian’s skin bacterial community. The ordination used for host species and Bd status contains all individuals sampled (*k* = 3, stress = 0.15), and the ordination used to compare sampling sites only includes green frogs (*k* = 3, stress = 0.12), the only species to be sampled at more than two sites. PERMANOVA results indicated that sampling site and species (*p* < 0.05) but not Bd status (*p* > 0.05) influenced bacterial community beta diversity.

Bray–Curtis, Jaccard, unweighted Unifrac, and weighted Unifrac yielded consistent results, therefore only Bray–Curtis results are presented for simplicity. Host species (Pseudo-F = 5.34, df = 5, *R*^2^ = 0.26, *p* = 0.001) was significantly associated with bacterial beta diversity differences among individuals (Figure 3). Compositional differences among frog host species’ microbiota were significant between bullfrogs and green frogs, bullfrogs and spring peepers, and wood frogs and green frogs (pairwise PERMANOVA: *p* < 0.05). There were also site-level differences in beta diversity among the three green frog populations (Figure 3; PERMANOVA – Pseudo-F = 4.28, df = 2, *R*^2^ = 0.25, *p* = 0.001).

**FIGURE 4:**
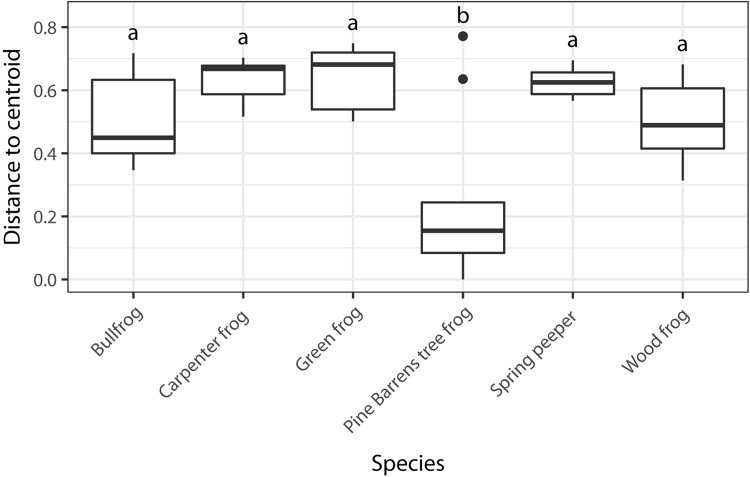
Dispersion among skin bacterial communities of each of the six species of frogs sampled based on Bray–Curtis dissimilarity matrix. Different letters represent species with significant differences in group dispersion based on a permutational test of dispersion (FDR-corrected *p* < 0.05).

I found significant differences in beta dispersion (distance to centroid) among host species (*F*_5,70_ = 14.03, *p* = 0.001). These differences were driven by the relatively small distance to centroid observed among Pine Barrens treefrogs (Figure 4), indicating that Pine Barrens treefrogs’ skin bacterial communities were more compositionally similar to one another than individuals of other species. This pattern is also evident in the NMDS plots of Bray–Curtis distances (Figure 3), where Pine Barrens treefrog individuals cluster closely in ordination space and have a relatively narrow confidence ellipse.

### Bd Prevalence

No clinical signs of chytridiomycosis were observed during frog swabbing. Nine individuals tested positive for Bd (Table 1 and Supplementary Table S1), making Bd prevalence 11.8% across all individuals sampled (*n* = 76). One individual, a green frog at Morin Pond, yielded an inconclusive result with only one of three PCR wells testing positive for Bd. Because there was not enough DNA to re-test this individual, it was conservatively considered negative for Bd.

Bacterial alpha diversity did not differ between Bd-positive and Bd-negative individuals (KW – OTU richness: χ^2^ = 1.89, *p* = 0.17; Chao1: χ^2^ = 2.02, *p* = 0.15). Among spring peepers, the only species with roughly equal numbers of Bd positive (*n* = 4) and negative (*n* = 3) individuals, there were also no differences in alpha diversity based on Bd status (KW – OTU richness: χ^2^ = 0.8, *p* = 0.37; Chao1: χ^2^ = 1.13, *p* = 0.29), suggesting that this trend is not simply an artifact of unbalanced sample sizes. Using Pearson correlation, I did not find that either alpha diversity metric correlated with Bd infection intensity (Supplementary Table S1; OTU richness: *R* = 0.11, *p* = 0.34; Chao1: *R* = 0.11, *p* = 0.33).

Bd status explained very little variation in community structure and composition and was not significant in PERMANOVA results based on Bray–Curtis dissimilarities (Pseudo-F = 1.17, df = 1, *R*^2^ = 0.02, *p* = 0.75). Furthermore, Bd-positive individuals were nested within overall community structure in ordination space (Figure 3). Taken together, these results indicate that differences among skin bacterial communities were not related to Bd status.

### Comparison to Antifungal Isolates Database

Of the 2194 frog-associated OTUs, 11.9% (260) matched inhibitory isolates in the antifungal isolate database. These putative anti-Bd OTUs made up 32.6% of bacterial abundance within host-associated bacterial communities. The mean number of anti-Bd OTUs across individuals was 21 (SD ±14), and the majority (80%) of anti-Bd OTUs were classified as Proteobacteria, although members of Bacteroidetes, Actinobacteria, and Firmicutes were also present.

**FIGURE 5:**
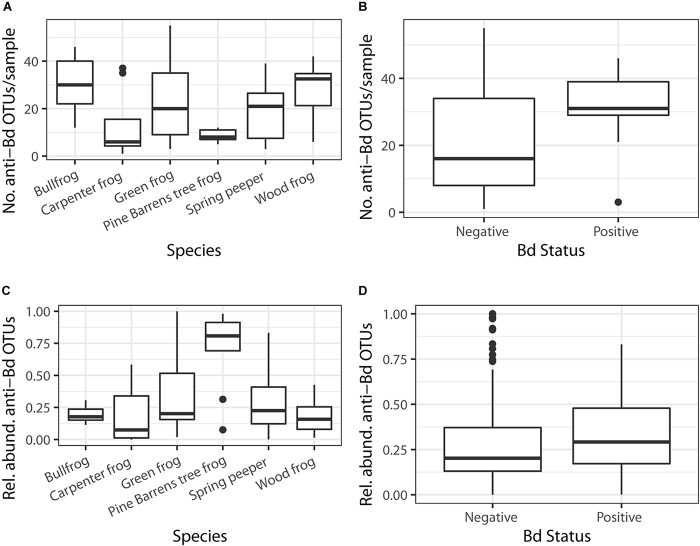
The number of putative anti-Bd OTUs varied based on **(A)** host species and **(B)** Bd status (GLMM *p* < 0.05). There were no differences in putative anti-Bd OTUs across **(C)** host species or **(D)** Bd status once relative abundances of bacteria were considered (GLMM *p* > 0.05).

**FIGURE 6:**
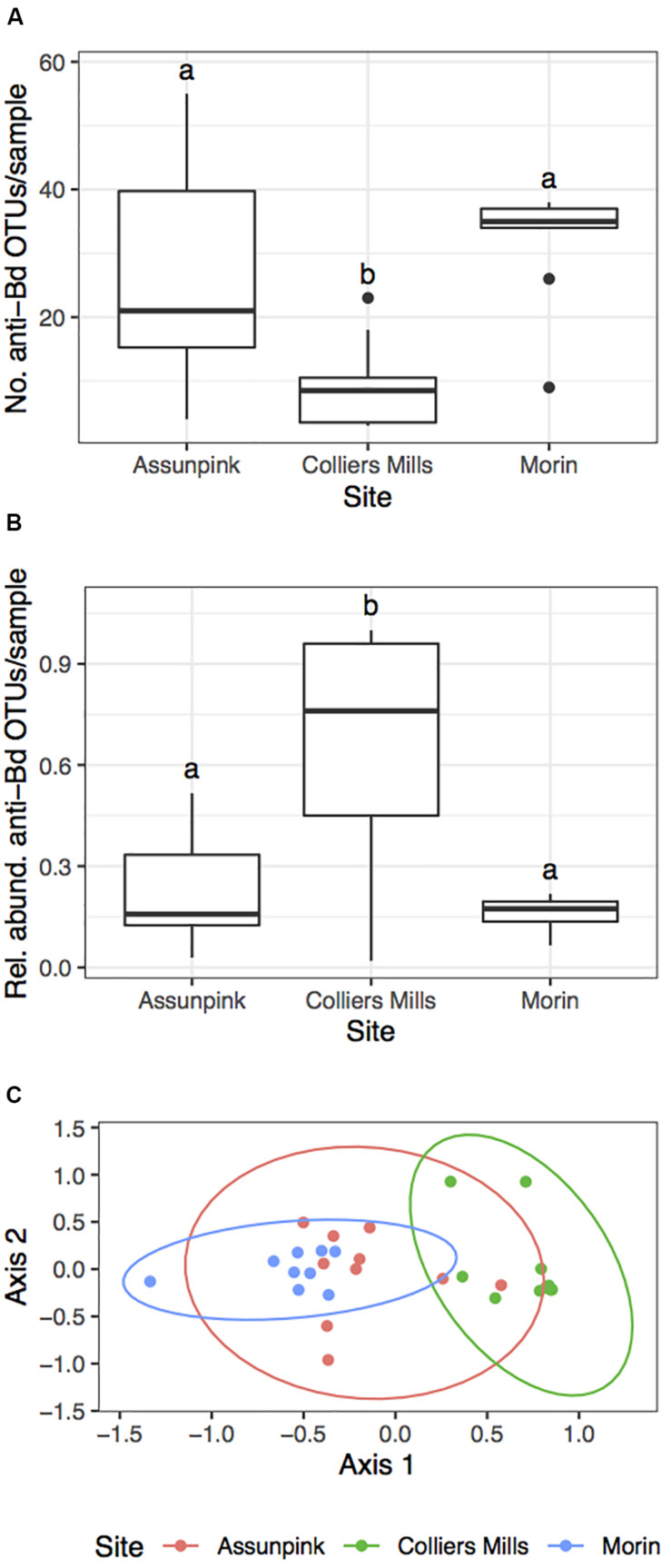
Summary of putative anti-Bd OTU community metrics among three green frog populations. The **(A)** number, **(B)** relative abundance, and **(C)** beta diversity (NMDS: *k* = 2, stress = 0.151) of putative anti-Bd OTUs varied significantly among green frog populations. Letters in **(A)** and **(B)** denote significant differences between sites (FDR-corrected *p* < 0.05). Pairwise PERMANOVAs indicated that there were significant differences in anti-Bd beta diversity between all site pairs (*p* < 0.05).

There were significant differences in the number of anti-Bd OTUs across host species and between Bd-positive and Bd-negative individuals (Figure 5; GLMM – Species: χ^2^ = 39.6, df = 5, *p* < 0.001; Bd status: χ^2^ = 27.5, df = 1, *p* < 0.001). However, these differences were no longer significant when considering the relative abundance of anti-Bd OTUs among host species and between Bd-positive and Bd-negative individuals (Figure 5; GLMM – Species: χ^2^ = 6.40, df = 5, *p* = 0.27; Bd status: χ^2^ = 0.37, df = 1, *p* = 0.54), suggesting that OTU richness is not always related to abundance. There were significant differences in both the number (KW χ^2^ = 12.81, *p* = 0.002) and relative abundance (KW χ^2^ = 10.67, *p* = 0.005) of anti-Bd OTUs across the three green frog populations (Figure 6).

PERMANOVA results indicated that host species identity (Pseudo-F = 4.25, df = 5, *R*^2^ = 0.21, *p* = 0.001) significantly influenced anti-Bd community beta diversity (Supplementary Figure S2). Additionally, there were significant site-level differences in putative anti-Bd OTUs among the three populations of green frogs (Figure 6; PERMANOVA – Pseudo-F = 4.79, df = 2, *R*^2^ = 0.27, *p* = 0.001). However, Bd status had no significant effect on anti-Bd community beta diversity (Pseudo-F = 1.12, df = 1, *R*^2^ = 0.015, *p* = 0.8). Similar to the overall beta diversity results, there were significant differences in beta dispersion for anti-Bd OTU communities among host species (*F*_5,70_ = 10.4, *p* = 0.001). Once again, these differences were driven by the relatively small dispersion among Pine Barrens treefrogs. These trends suggest that patterns in anti-Bd community diversity reflect patterns in overall community diversity.

### Indicator Species

**FIGURE 7:**
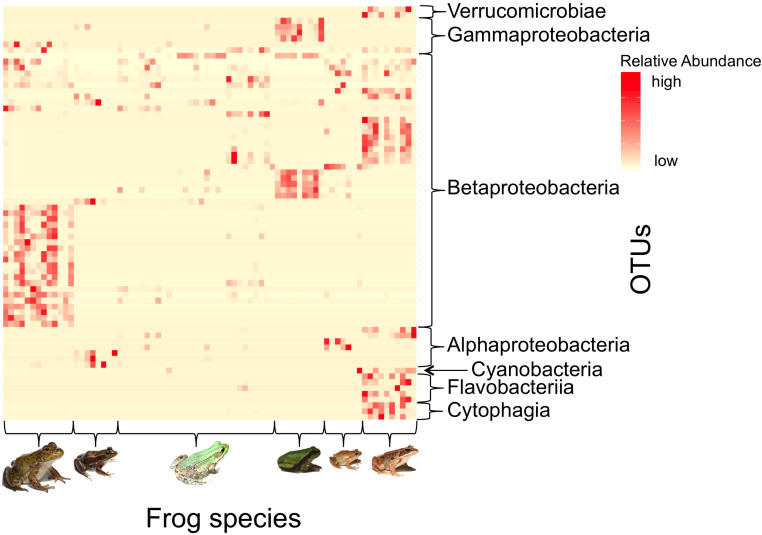
Heatmap depicting the relative abundances of indicator bacterial taxa (IndVal > 0.7) across frog host species. Each row depicts a unique indicator OTU (Class taxonomic level displayed) and each column indicates an individual frog (Frog species from left to right: American bullfrog, carpenter frog, green frog, Pine Barrens treefrog, spring peepers, and wood frogs). There were 71 OTUs (see Supplementary Table S2) that were strong indicators for at least one frog species, and most indicators were unique among frog species. [Photo credits: Bullfrog, spring peeper, and wood frog – Brian Gratwicke; Carpenter frog – Brady Beck; Green frog – Peter J. Morin; Pine Barrens treefrog – AK].

There were 71 OTUs strongly associated (IndVal > 0.7) with at least one frog species (Figure 7). Of these indicator taxa, 30 (42.3%) were putative anti-Bd OTUs, which is significantly more than expected based on the proportion of putative anti-Bd OTUs among the 2194 OTUs in the larger dataset (χ^2^ = 62.94, df = 2, *p* < 0.001). The majority (84.5%) of indicator OTUs belonged to the phylum Proteobacteria. Only 13 of all indicators were associated with more than one frog species (Supplementary Table S2).

In the indicator analysis based on host Bd status, six OTUs were highly associated with Bd*-*positive individuals. All six OTUs belonged to the family Comamonadaceae (Phylum: Proteobacteria, Class: Betaproteobacteria, Order: Burkholderiales). Three out of six of these OTUs were putative anti-Bd isolates based on consensus matches in the antifungal isolate database, indicating that several potential anti-Bd OTUs were strongly associated with Bd-positive individuals.

### Comparison to Environment

Within the environmental samples, there were no significant differences in OTU richness (KW χ^2^ = 1.83, *p* = 0.77) or Chao1 index (KW χ^2^ = 2.75, *p* = 0.6) among sites. Mean OTU richness was significantly higher in environmental samples compared to frog samples (KW χ^2^ = 4.68, *p* = 0.03), but there were no differences in Chao1 index (KW χ^2^ = 3.8, *p* = 0.051). Beta diversity was significantly different between environmental and frog samples (PERMANOVA – Pseudo-F = 1.63, df = 1, *R*^2^ = 0.02, *p* = 0.02) when samples were pooled across species and sites. Taken together, these results suggest differences in community structure between frog and environmental bacterial communities.

## Discussion

In this survey of skin microbiota of six species of amphibians distributed across multiple sites, I found that host species and sampling site influenced bacterial alpha and beta diversity and that host-associated bacterial communities differed from environmental bacterial communities. These results provide additional evidence that amphibian skin microbial communities tend to be host species-specific (McKenzie et al., 2012; Kueneman et al., 2014; Walke et al., 2014), compositionally distinct from the environment (Loudon et al., 2014; Walke et al., 2014; Rebollar et al., 2016b), and can vary among sites (Kueneman et al., 2014; Hughey et al., 2017; Muletz-Wolz et al., 2017a, b). While Bd status was not related to overall differences in bacterial alpha and beta diversity, Bd-positive individuals harbored significantly more putative anti-Bd OTUs than Bd-negative individuals, and indicator species analysis showed that putative anti-Bd OTUs were integral members of skin bacterial communities across species. These results suggest that species living with asymptomatic Bd infection may be benefiting from the anti-Bd function of host-associated bacterial communities.

It is difficult to disentangle the cause and effect relationship between microbiota influencing Bd susceptibility and Bd infection impacting microbiota structure, but experimental evidence suggests that both outcomes are possible and not mutually exclusive (Voyles et al., 2009; Jani and Briggs, 2014; Walke et al., 2015). I hypothesized that Bd-positive and Bd-negative individuals would harbor distinct bacterial communities because of the previously documented connection between Bd and host microbiota, but I did not find evidence to support this. However, there were relatively few Bd-positive individuals (9/76), and these individuals were spread across multiple species and sites. In addition, despite roughly equal numbers of Bd-positive and Bd-negative spring peepers, there were still no observable differences in the skin bacterial communities of these individuals. Belden et al. (2015) also found no link between bacterial community structure and Bd infection status, and Longo et al. (2015) found evidence that environmental variables were more important than Bd status in structuring skin microbial communities among species with enzootic Bd infections, suggesting that the amount of time the population has experienced Bd (enzootic vs. epizootic infection) could influence the relationship between microbial community structure and Bd infection. Because environmental and temporal factors likely influence this relationship (Estrada et al., 2019), it is possible that the limited seasonal sampling in the present study missed important variation in Bd presence and microbial community structure. As such, studies examining the microbiota–Bd relationship in populations across time are needed to further elucidate these dynamics.

Furthermore, microbiota–Bd interactions may vary depending on host species susceptibility to Bd. For example, if a species tolerates Bd infection due to other components of their innate defenses (Rollins-Smith et al., 2011), Bd infection may not drastically affect skin microbial community structure. Additionally, one limitation of Bd testing is that a positive detection using qPCR does not necessarily equate to an active or viable Bd infection. Several species sampled in this study, such as spring peepers, green frogs, and bullfrogs, are known to live with Bd asymptomatically (Gahl et al., 2012), making observation of clinical signs of Bd infection in the field difficult. Bullfrogs are believed to serve as reservoirs of Bd infection (Daszak et al., 2004) and can transfer Bd to wood frogs, a species that is susceptible to mortality from Bd (Greenspan et al., 2013). To my knowledge, the susceptibility of carpenter frogs and Pine Barrens treefrogs to Bd is unknown. Asymptomatic carriers of Bd may be able to persist with Bd infection due to host-associated microbial communities that are dominated by anti-Bd bacteria, which could prevent amphibian hosts from developing disease.

While I did not find a clear relationship between overall bacterial community structure and Bd status, several patterns emerged among putative anti-Bd bacterial communities. Using a reference antifungal isolate database (Woodhams et al., 2015), 11.8% of OTUs were classified as potential anti-Bd bacteria, and these OTUs comprised nearly a third of total bacterial abundance across samples. Furthermore, significantly more indicator OTUs were putative anti-Bd taxa than would be expected based on the proportion of anti-Bd OTUs among all frog OTUs. This result suggests that anti-Bd OTUs are integral members of bacterial communities based on their prevalence and abundance across individuals.

The community structure (i.e., beta diversity) of the anti-Bd bacterial community varied among host species, demonstrating that taxonomically unique antifungal bacterial communities occur across hosts. Among the green frogs, the only species sampled across three sites, there were also significant differences in the anti-Bd bacterial community structure among populations. Using a culture-based approach, I previously found that green frogs at these sites harbored unique communities of anti-Bd bacteria (Kruger, 2020), a result that has now been similarly demonstrated using the 16S rRNA sequencing methods of the present study. Previous studies have also documented the presence of distinct communities of anti-Bd bacteria among localities (Lam et al., 2010; Flechas et al., 2012; Barnes et al., 2020). The presence of novel communities of anti-Bd bacteria across sites coupled with the high prevalence of anti-Bd taxa among indicator OTUs suggest that amphibians may opportunistically acquire potentially beneficial bacteria rather than rely upon a distinct subset of bacterial taxa that are common across sites.

Bd-positive individuals harbored significantly more anti-Bd OTUs than Bd-negative individuals. This result is opposite from what might be expected if individuals with more anti-Bd OTUs are less likely to be susceptible to Bd due to bacterial inhibition of Bd growth. However, Muletz-Wolz et al. (2019) also found an increase in the abundance of anti-Bd bacteria on amphibians after Bd infection, and Walke et al. (2017) found more anti-Bd OTUs among populations with higher Bd prevalence. This pattern may be due to selection for anti-Bd bacteria after Bd colonizes the skin as a mechanism to fight infections (Walke et al., 2017) or to cutaneous disruption from Bd infection leading to a more favorable environment for some anti-Bd bacteria (Muletz-Wolz et al., 2019). Alternatively, Bd presence at sites may have already selected for frogs that harbor more anti-Bd bacteria (Belden et al., 2015), as Bd-positive individuals without these bacteria may have experienced Bd-related mortality. While resistance to Bd is likely also related to other aspects of host innate immunity (Rollins-Smith et al., 2011), these results suggest that anti-Bd OTUs may be playing a role in amphibian resistance to Bd in these populations.

Patterns in anti-Bd OTU relative abundance did not reflect patterns in anti-Bd OTU richness. Differences between Bd positive and negative individuals were no longer present when anti-Bd OTU relative abundances were considered, and could imply that the increase in anti-Bd OTU richness among Bd-positive individuals does not translate to an increase in anti-Bd function if function is better predicted by dominance rather than OTU richness. Because Bd prevalence was low among most of the species sampled here, there may not be strong selection for anti-Bd bacteria to dominate these communities (Walke et al., 2017). Rather, the presence of some anti-Bd OTUs may allow individuals to persist asymptomatically with low infection burden instead of eliminating infection entirely (Bresciano et al., 2015). This could be problematic if individuals remain reservoirs of infection and continue to transmit Bd to co-occurring amphibians that are more susceptible to mortality from Bd.

Finally, indicator analysis showed that there was a strong association between Bd-positive individuals and putative anti-Bd bacteria. Three out of six indicator OTUs for Bd-positive individuals were identified as potentially anti-Bd taxa. All six indicator OTUs belong to the family Comamonadaceae, which has previously been noted for having members with anti-Bd properties (Becker et al., 2015b; Woodhams et al., 2015). There is also evidence to suggest that frogs harboring bacteria from this group may be better equipped to clear Bd infection (Becker et al., 2015a). Walke et al. (2015) found that the relative abundance of a Comamonadaceae OTU was significantly higher in Bd-exposed frogs compared to non-exposed frogs and proposed that this shift in abundance was due to selection for this OTU after Bd exposure. My results provide supporting evidence that Comamonadaceae could play an important role in regulating Bd infection in amphibians.

The anti-Bd OTUs analyzed here were classified as potentially Bd-inhibitory based on 16S sequence similarity to bacteria that have been previously tested for Bd inhibition (Woodhams et al., 2015). While this approach to predicting function has been used in the past (Bletz et al., 2017b; Kueneman et al., 2017; Muletz-Wolz et al., 2019), 16S sequence similarity does not always indicate similar levels of Bd inhibition (Becker et al., 2015b). Despite its limitations, this approach allows for functional predictions of amphibian skin microbial communities and may help detect beneficial bacteria for future study. Given the interest in probiotic therapies against Bd (Bletz et al., 2013), results from assessments of wild amphibian populations should be used to help inform probiotic selection because they identify groups of bacteria that are integral members of host-associated microbial communities.

As the prevalence of emerging infectious diseases increases, it is important to identify the key drivers of host-associated microbial community structure and function in systems where microbes may play a role in disease resistance. The findings of this study suggest that anti-Bd bacteria may play an important role in host defenses among amphibians that are coexisting with Bd. Elucidating patterns in anti-Bd bacteria among host species may help inform use of conservation resources to prevent Bd spread or mitigate the effects of disease. However, exploration of other aspects of host immunity, such as antimicrobial peptides (Rollins-Smith et al., 2002), is also needed to provide insight on the mechanisms that allow some species to persist with Bd without developing chytridiomycosis. As such, studies focusing on identifying patterns in a single aspect of host immunity (e.g., microbial community composition *or* antimicrobial peptides) may not be sufficient to understand patterns in Bd infection. A more comprehensive approach to studying amphibian immune defenses may be crucial to discovering ways to ameliorate the spread of infectious disease and prevent mortality of at-risk amphibians.

## Data Availability Statement

Sequencing data submitted to the SRA database under Bioproject PRJNA601697 (https://www.ncbi.nlm.nih.gov/bioproject/PRJN A601697).

## Ethics Statement

The animal study was reviewed and approved by Rutgers University’s Institutional Animal Care and Use Committee.

## Author Contributions

AK is the sole author of this work.

## Conflict of Interest

The author declares that the research was conducted in the absence of any commercial or financial relationships that could be construed as a potential conflict of interest.
