# Supplementary material for: Frog Skin Microbiota Vary With Host Species and Environment but Not Chytrid Infection
Source: Front Microbiol. 2020 Jun 24;11:1330. doi: 10.3389/fmicb.2020.01330 (PMC7328345; doi:10.3389/fmicb.2020.01330)
Supplement: Supplementary file 1 [file Data_Sheet_1.docx]

Supplementary Material

# Supplementary Figures

**Supplementary Figure 1.** Map of sampling sites in New Jersey. ALB = Albertson Bog, CM = Colliers Mills WMA, AS = Assunpink WMA, MD = Morin Pond, and KAI = Kai Pond. Morin Pond and Kai Pond are located approximately 0.5 miles apart from each other, and are therefore listed together on the map.

**Supplementary Figure 2.** NMDS ordination of putative anti-Bd bacterial communities across sites and species based on Bray-Curtis dissimilarity matrix (k = 3, stress = 0.16). PERMANOVA results indicated that host species significantly influenced anti-Bd bacterial community beta-diversity (p < 0.05). The effect of site was determined by comparing the anti-Bd bacterial community among the three green frog populations (Figure 6c).

# Supplementary Tables

**Supplementary Table 1.** Summary of Bd qPCR data among the individuals (n = 9) that tested positive for Bd. “Wells tested” and “wells positive” refer to the number of replicate wells tested in qPCR assays, and the number of those wells that yielded a positive detection of Bd, respectively. Mean Ct and mean Bd load (= zoospore equivalents) are calculated as the average values of technical (positive) replicates. Sampling details are provided in Table 1, and qPCR details are provided in the main text.

| **Sample ID** | **Frog Species** | **Site** | **Wells positive** | **Wells tested** | **min Ct** | **max Ct** | **mean Ct** | **mean Bd load** |
| --- | --- | --- | --- | --- | --- | --- | --- | --- |
| AS.BF4 | Bullfrog | Assunpink | 3 | 3 | 34.35 | 34.81 | 34.51 | 21 |
| AS.BF7 | Bullfrog | Assunpink | 3 | 3 | 34.21 | 36.82 | 35.66 | 12.67 |
| AS.GF1 | Green frog | Assunpink | 3 | 6 | 21.58 | 39.63 | 33.42 | 46754.7 |
| Kai.SP2 | Spring peeper | Kai | 3 | 3 | 36.12 | 38.52 | 37.04 | 6 |
| Kai.SP3 | Spring peeper | Kai | 2 | 3 | 36.72 | 36.94 | 36.83 | 5.5 |
| Kai.SP4 | Spring peeper | Kai | 3 | 3 | 34.44 | 35.07 | 34.74 | 23 |
| Kai.SP6 | Spring peeper | Kai | 2 | 3 | 37.36 | 38.37 | 37.87 | 3 |
| MD.WF6 | Wood frog | Morin Pond | 3 | 3 | 34.3 | 35.29 | 34.78 | 32.3 |
| MD.WF8 | Wood frog | Morin Pond | 4 | 6 | 37.13 | 39.79 | 38.41 | 2 |

**Supplementary Table 2.** List of indicator OTUs found on frog skin, including the OTU ID, IndVal statistic, p-value indicating significance, and frog host species for which the OTUs were considered significant (IndVal > 0.7, p < 0.05) indicators. OTUs marked with an asterisk (*) had consensus matches in the antifungal isolate database (Woodhams et al., 2015) at a 97% sequence similarity threshold.

| OTU | Indval-stat | p-value | host species | Taxonomy |
| --- | --- | --- | --- | --- |
| 974797 | 0.739 | 0.009 | bullfrog+green | k__Bacteria p__Proteobacteria c__Betaproteobacteria o__Burkholderiales f__Comamonadaceae g__ s__ |
| 798634* | 0.8 | 0.003 | bullfrog+green+peeper | k__Bacteria p__Proteobacteria c__Betaproteobacteria o__Burkholderiales f__Comamonadaceae NA NA |
| 672144* | 0.759 | 0.005 | bullfrog+green+peeper+wood | k__Bacteria p__Proteobacteria c__Betaproteobacteria o__Burkholderiales f__Comamonadaceae g__ s__ |
| 818450* | 0.705 | 0.024 | bullfrog+green+wood | k__Bacteria p__Proteobacteria c__Betaproteobacteria o__Burkholderiales f__Comamonadaceae g__ s__ |
| 576785* | 0.795 | 0.002 | bullfrog+peeper+wood | k__Bacteria p__Proteobacteria c__Betaproteobacteria o__Burkholderiales f__Comamonadaceae g__ s__ |
| 576928* | 0.874 | 0.001 | bullfrog+peeper+wood | k__Bacteria p__Proteobacteria c__Betaproteobacteria o__Burkholderiales f__Comamonadaceae g__ s__ |
| 578572* | 0.784 | 0.001 | bullfrog+peeper+wood | k__Bacteria p__Proteobacteria c__Betaproteobacteria o__Burkholderiales f__Comamonadaceae g__ s__ |
| 614969* | 0.721 | 0.005 | bullfrog+wood | k__Bacteria p__Proteobacteria c__Betaproteobacteria o__Rhodocyclales f__Rhodocyclaceae g__ s__ |
| 222554 | 0.92 | 0.001 | bullfrog | k__Bacteria p__Proteobacteria c__Betaproteobacteria o__Methylophilales f__Methylophilaceae g__ s__ |
| 306546 | 0.877 | 0.001 | bullfrog | k__Bacteria p__Proteobacteria c__Betaproteobacteria o__Methylophilales f__Methylophilaceae g__ s__ |
| 312987 | 0.832 | 0.001 | bullfrog | k__Bacteria p__Proteobacteria c__Betaproteobacteria o__Methylophilales f__Methylophilaceae g__Methylotenera s__mobilis |
| 322972 | 0.999 | 0.001 | bullfrog | k__Bacteria p__Proteobacteria c__Betaproteobacteria o__Methylophilales f__Methylophilaceae g__ s__ |
| 661442* | 0.7 | 0.002 | bullfrog | k__Bacteria p__Proteobacteria c__Betaproteobacteria o__Burkholderiales f__Comamonadaceae g__ s__ |
| 777498 | 0.95 | 0.001 | bullfrog | k__Bacteria p__Proteobacteria c__Betaproteobacteria o__Methylophilales f__Methylophilaceae g__Methylotenera s__mobilis |
| 4315164 | 0.704 | 0.005 | bullfrog | k__Bacteria p__Proteobacteria c__Betaproteobacteria o__Burkholderiales f__Comamonadaceae NA NA |
| 4397450 | 0.723 | 0.001 | bullfrog | k__Bacteria p__Proteobacteria c__Gammaproteobacteria o__Methylococcales f__Crenotrichaceae g__Crenothrix s__ |
| New.CleanUp.ReferenceOTU0* | 0.806 | 0.001 | bullfrog | k__Bacteria p__Proteobacteria c__Betaproteobacteria o__Burkholderiales f__Comamonadaceae g__ s__ |
| New.ReferenceOTU1509 | 0.734 | 0.001 | bullfrog | k__Bacteria p__Proteobacteria c__Betaproteobacteria o__Methylophilales f__Methylophilaceae g__Methylotenera s__mobilis |
| New.ReferenceOTU1536* | 0.83 | 0.001 | bullfrog | k__Bacteria p__Proteobacteria c__Betaproteobacteria o__Burkholderiales f__Comamonadaceae g__Paucibacter s__ |
| New.ReferenceOTU1543* | 0.73 | 0.001 | bullfrog | k__Bacteria p__Proteobacteria c__Betaproteobacteria o__Burkholderiales f__Comamonadaceae NA NA |
| New.ReferenceOTU165 | 0.784 | 0.001 | bullfrog | k__Bacteria p__Proteobacteria c__Betaproteobacteria o__Methylophilales f__Methylophilaceae g__ s__ |
| New.ReferenceOTU251 | 0.784 | 0.001 | bullfrog | k__Bacteria p__Proteobacteria c__Betaproteobacteria o__Burkholderiales f__Comamonadaceae g__ s__ |
| New.ReferenceOTU3830* | 0.784 | 0.001 | bullfrog | k__Bacteria p__Proteobacteria c__Betaproteobacteria o__Burkholderiales f__Comamonadaceae g__ s__ |
| New.ReferenceOTU3981 | 0.734 | 0.001 | bullfrog | k__Bacteria p__Proteobacteria c__Betaproteobacteria o__Burkholderiales f__Comamonadaceae g__ s__ |
| New.ReferenceOTU4424* | 0.797 | 0.001 | bullfrog | k__Bacteria p__Proteobacteria c__Betaproteobacteria o__Burkholderiales f__Comamonadaceae g__ s__ |
| New.ReferenceOTU4804 | 0.734 | 0.001 | bullfrog | k__Bacteria p__Proteobacteria c__Betaproteobacteria o__Methylophilales f__Methylophilaceae g__ s__ |
| New.ReferenceOTU5397 | 0.734 | 0.001 | bullfrog | k__Bacteria p__Proteobacteria c__Betaproteobacteria o__Methylophilales f__Methylophilaceae g__ s__ |
| New.ReferenceOTU6735 | 0.783 | 0.002 | bullfrog | k__Bacteria p__Proteobacteria c__Betaproteobacteria o__Burkholderiales f__Comamonadaceae g__ s__ |
| New.ReferenceOTU73 | 1 | 0.001 | bullfrog | k__Bacteria p__Proteobacteria c__Betaproteobacteria o__Methylophilales f__Methylophilaceae g__ s__ |
| New.ReferenceOTU81* | 0.944 | 0.001 | bullfrog | k__Bacteria p__Proteobacteria c__Betaproteobacteria o__Burkholderiales f__Comamonadaceae g__Paucibacter s__ |
| 2912622* | 0.745 | 0.003 | carpenter+pb_treefrog | k__Bacteria p__Proteobacteria c__Betaproteobacteria o__Neisseriales f__Neisseriaceae g__Chromobacterium s__ |
| 330923 | 0.798 | 0.001 | carpenter+wood | k__Bacteria p__Proteobacteria c__Betaproteobacteria o__Burkholderiales f__Comamonadaceae g__Rhodoferax s__ |
| 107461 | 0.776 | 0.001 | carpenter_frog | k__Bacteria p__Proteobacteria c__Alphaproteobacteria o__Rhizobiales f__ g__ s__ |
| 749696 | 0.7 | 0.003 | carpenter_frog | k__Bacteria p__Proteobacteria c__Alphaproteobacteria o__Sphingomonadales f__Sphingomonadaceae g__ s__ |
| New.ReferenceOTU1022 | 0.707 | 0.001 | carpenter_frog | k__Bacteria p__Proteobacteria c__Alphaproteobacteria o__Sphingomonadales f__Sphingomonadaceae g__Novosphingobium s__ |
| New.ReferenceOTU6 | 0.769 | 0.001 | carpenter_frog | k__Bacteria p__Proteobacteria c__Betaproteobacteria NA NA NA NA |
| New.ReferenceOTU0* | 0.868 | 0.001 | green+pb_treefrog+peeper | k__Bacteria p__Proteobacteria c__Betaproteobacteria o__Burkholderiales f__Alcaligenaceae g__Achromobacter s__ |
| 38733 | 0.796 | 0.001 | peeper+wood | k__Bacteria p__Cyanobacteria c__Chloroplast o__Stramenopiles f__ g__ s__ |
| 719367 | 0.831 | 0.001 | peeper+wood | k__Bacteria p__Proteobacteria c__Betaproteobacteria o__Burkholderiales f__Comamonadaceae g__ s__ |
| 104044* | 0.882 | 0.001 | Pine_Barrens_tree_frog | k__Bacteria p__Proteobacteria c__Gammaproteobacteria o__Enterobacteriales f__Enterobacteriaceae g__ s__ |
| 228065* | 0.951 | 0.001 | Pine_Barrens_tree_frog | k__Bacteria p__Proteobacteria c__Betaproteobacteria o__Burkholderiales f__Alcaligenaceae g__Achromobacter s__ |
| 558264* | 0.817 | 0.001 | Pine_Barrens_tree_frog | k__Bacteria p__Proteobacteria c__Betaproteobacteria o__Burkholderiales f__Alcaligenaceae g__Achromobacter s__ |
| 922761 | 1 | 0.001 | Pine_Barrens_tree_frog | k__Bacteria p__Proteobacteria c__Gammaproteobacteria o__Enterobacteriales f__Enterobacteriaceae NA NA |
| 4403542* | 0.924 | 0.001 | Pine_Barrens_tree_frog | k__Bacteria p__Proteobacteria c__Gammaproteobacteria o__Enterobacteriales f__Enterobacteriaceae g__ s__ |
| 4451045* | 0.97 | 0.001 | Pine_Barrens_tree_frog | k__Bacteria p__Proteobacteria c__Betaproteobacteria o__Burkholderiales f__Alcaligenaceae g__Achromobacter s__ |
| New.ReferenceOTU1763 | 0.87 | 0.001 | Pine_Barrens_tree_frog | k__Bacteria p__Proteobacteria c__Betaproteobacteria o__Burkholderiales f__Alcaligenaceae g__ s__ |
| New.ReferenceOTU6067 | 0.816 | 0.001 | Pine_Barrens_tree_frog | k__Bacteria p__Proteobacteria c__Gammaproteobacteria o__Enterobacteriales f__Enterobacteriaceae g__Erwinia s__ |
| New.ReferenceOTU72 | 0.926 | 0.001 | Pine_Barrens_tree_frog | k__Bacteria p__Proteobacteria c__Betaproteobacteria o__Burkholderiales f__Alcaligenaceae g__ s__ |
| 146007 | 0.731 | 0.001 | spring_peeper | k__Bacteria p__Proteobacteria c__Alphaproteobacteria o__Sphingomonadales f__Sphingomonadaceae g__Sphingomonas s__ |
| 734945 | 0.751 | 0.001 | spring_peeper | k__Bacteria p__Proteobacteria c__Alphaproteobacteria o__Sphingomonadales f__Sphingomonadaceae g__Sphingomonas s__echinoides |
| 822419* | 0.806 | 0.001 | spring_peeper | k__Bacteria p__Proteobacteria c__Betaproteobacteria o__Burkholderiales f__Oxalobacteraceae g__ s__ |
| 230812* | 0.775 | 0.001 | Wood_frog | k__Bacteria p__Bacteroidetes c__Flavobacteriia o__Flavobacteriales f__Flavobacteriaceae g__Flavobacterium s__ |
| 278075 | 0.805 | 0.001 | Wood_frog | k__Bacteria p__Proteobacteria c__Alphaproteobacteria o__Sphingomonadales f__Sphingomonadaceae g__Zymomonas s__ |
| 511461* | 0.707 | 0.001 | Wood_frog | k__Bacteria p__Bacteroidetes c__Flavobacteriia o__Flavobacteriales f__Flavobacteriaceae g__Flavobacterium s__ |
| 584580* | 0.71 | 0.002 | Wood_frog | k__Bacteria p__Proteobacteria c__Betaproteobacteria o__Burkholderiales f__Comamonadaceae g__Hydrogenophaga s__ |
| 585360 | 0.707 | 0.001 | Wood_frog | k__Bacteria p__Verrucomicrobia c__Verrucomicrobiae o__Verrucomicrobiales f__Verrucomicrobiaceae g__Prosthecobacter NA |
| 659078 | 0.894 | 0.001 | Wood_frog | k__Bacteria p__Bacteroidetes c__Cytophagia o__Cytophagales f__Cytophagaceae g__Flectobacillus s__ |
| 699027* | 0.742 | 0.001 | Wood_frog | k__Bacteria p__Bacteroidetes c__Flavobacteriia o__Flavobacteriales f__Flavobacteriaceae g__Flavobacterium s__ |
| 886673* | 0.773 | 0.001 | Wood_frog | k__Bacteria p__Bacteroidetes c__Flavobacteriia o__Flavobacteriales f__Flavobacteriaceae g__Flavobacterium s__succinicans |
| 971457 | 0.775 | 0.001 | Wood_frog | k__Bacteria p__Bacteroidetes c__Flavobacteriia o__Flavobacteriales f__Flavobacteriaceae g__Flavobacterium s__ |
| 972719 | 0.707 | 0.001 | Wood_frog | k__Bacteria p__Bacteroidetes c__Cytophagia o__Cytophagales f__Cytophagaceae g__Flectobacillus s__ |
| 998905 | 0.892 | 0.001 | Wood_frog | k__Bacteria p__Proteobacteria c__Alphaproteobacteria o__Caulobacterales f__Caulobacteraceae g__Mycoplana s__ |
| 3480259 | 0.996 | 0.001 | Wood_frog | k__Bacteria p__Bacteroidetes c__Cytophagia o__Cytophagales f__Cytophagaceae g__Emticicia s__ |
| 4306063* | 0.722 | 0.002 | Wood_frog | k__Bacteria p__Proteobacteria c__Betaproteobacteria o__Burkholderiales f__Comamonadaceae g__Hydrogenophaga s__ |
| 4329804* | 0.76 | 0.001 | Wood_frog | k__Bacteria p__Proteobacteria c__Betaproteobacteria o__Rhodocyclales f__Rhodocyclaceae g__ s__ |
| 4340146 | 0.888 | 0.001 | Wood_frog | k__Bacteria p__Proteobacteria c__Betaproteobacteria o__Burkholderiales f__Comamonadaceae g__ s__ |
| 4351336* | 0.834 | 0.001 | Wood_frog | k__Bacteria p__Proteobacteria c__Betaproteobacteria o__Burkholderiales f__Comamonadaceae g__Hydrogenophaga s__ |
| 4389007 | 0.707 | 0.001 | Wood_frog | k__Bacteria p__Verrucomicrobia c__Verrucomicrobiae o__Verrucomicrobiales f__Verrucomicrobiaceae g__Luteolibacter s__ |
| 4429445 | 0.774 | 0.001 | Wood_frog | k__Bacteria p__Proteobacteria c__Betaproteobacteria o__Burkholderiales f__Comamonadaceae g__Hydrogenophaga s__ |
| 4430221 | 0.768 | 0.001 | Wood_frog | k__Bacteria p__Proteobacteria c__Betaproteobacteria o__Burkholderiales f__Comamonadaceae g__Hydrogenophaga s__ |
| New.ReferenceOTU3568 | 0.707 | 0.001 | Wood_frog | k__Bacteria p__Proteobacteria c__Betaproteobacteria o__Burkholderiales f__Comamonadaceae g__ s__ |
